# Editorial: Feeding and nutritional strategies for sows and piglets to improve piglets' robustness

**DOI:** 10.3389/fvets.2023.1250805

**Published:** 2023-07-12

**Authors:** Diana Luise, Ester Arévalo Sureda

**Affiliations:** ^1^Department of Agricultural and Food Science, University of Bologna, Bologna, Italy; ^2^Nutrition and Animal-Microbiota EcoSystems Lab, Department of Biosystems, KU Leuven, Leuven, Belgium

**Keywords:** suckling and lactation period, post-weaning diarrhea, gut health, early life, antimicrobial abuse, gut maturation

Piglet mortality and morbidity during the pre- and post-weaning periods are still a big issue for the swine industry, causing economic and ethical concerns for farmers and consumers (1). The low robustness of young piglets has been attributed to several factors, such as the immaturity of the digestive and immune systems in early life; the use of hyperprolific sows' lines, with an increased number of low-body weight piglets and decreased capacity of the sows to wean all piglets; and management practices to improve sows' welfare. Thus, the improvement of piglets' health in early life is of utmost importance, and a key approach involves nutritional and feeding strategies. Consequently, this would also contribute to the reduction in the use of antibiotics and the replacement of the pharmacological levels of ZnO, which have all been linked to antimicrobial resistance and pollution.

To enhance piglet robustness, nutritional and feeding strategies can be implemented either directly on the piglets, or indirectly on the sows, both during the gestation and lactation periods (2). This Research Topic aimed to collect articles showing new feeding and nutritional strategies for sows and piglets to promote gut and immune system maturation of piglets and boost their health in early life.

The study by Li et al., presented an experiment evaluating the health benefits of maternal supplementation with nucleotides on resistance to infection (LPS challenge) in newborn piglets. The authors investigated the effects on the systemic immune response, as well as on intestinal histology and immune response at gene expression and protein levels of the newborn piglets 1 h after an LPS challenge. The results indicated that nucleotides' supplementation to sows had an anti-inflammatory systemic effect, seen as non-reactivity to LPS challenge, at both gene expression and protein levels. Besides, piglets born from nucleotide supplemented sows had increased villi height and goblet cell numbers in the small intestine. In conclusion, piglets' robustness at birth can be improved by nucleotides' supplementation to sows during the last period of gestation.

Nonetheless, support of piglet's health by sow supplementation seems to have lost popularity to a direct approach of piglet's supplementation. Most of the strategies to improve piglet's gut health and robustness in this research collection included feeding interventions to piglets during the suckling and post-weaning periods. Indeed, the focus of four of the articles included is to reduce post-weaning diarrhea, which remains a major source of mortality and morbidity in swine production.

Correa et al. focused their attention on the importance of milk supplements during the suckling period. Furthermore, it strongly suggested that not only the supplement of choice is of high importance, but also, that the administration system can have a significant impact. In this study, two commercially available piglet supplements similar in energy content, vitamins, minerals, and probiotic composition, with a difference in palatability due to the use of lactose in one of the milk supplements, and artificial sweetener in the other. Their findings included a reduced mortality during the first days of supplementation, as well as an increase in the activity of piglets by enhancement of social and curiosity behaviors. Additionally, milk supplementation with an automatic dispenser showed a reduction in competition for suckling after 10 days. Moreover, the microbiota ecosystem showed that supplementation with a lactose-containing milk supplement enriched the short-chain fatty acid producing bacteria. However, the effects of the milk supplements on microbiota were overruled after solid feed was introduced. Thus, it can be concluded that milk supplementation is more effective when delivered with automatic dispensers, and artificially sweetened milk supplements lead to worse outcomes than those containing sweetened whey.

The effects of feeding fermented liquid feed (FLF) based on fermented cereals supplemented with the probiotic *Pediococcus acidilactici* (*P. acidilactici*) to suckling and weaning piglets challenged with Enterotoxigenic *Escherichia coli* (ETEC) F4 at weaning was investigated by Xu et al.. Although the analysis of the FLF showed a reduction in pH and an increase in the abundance of *P. acidilactici*, this feeding strategy did not affect the performance neither the diarrhea score nor the immune response of piglets when started at 14 days of age. The results aligned with previous studies testing FLF for weaners (3, 4), further suggesting fermentation of only the cereal fraction to increase the ingestion of probiotics, despite its inability to counteract ETEC infection.

The study by Larsen et al., investigated the efficacy of transplantation of bacteria-free filtrate of feces (fecal filtrate transplant, FFT) derived from healthy lactating sows to early life piglets (1–6 days of life). FFT has shown promising safety potential as the risk of transferring pathogenic microorganisms (bacteria, fungi, protozoa) is partly eliminated, while bacteriophages are kept, which play a crucial role in the intestinal settlement in early life (5, 6). In this pilot study, the authors showed that early life FFT treatment can reduce the occurrence of post-weaning diarrhea and intestinal inflammation resulting in a lower hematopoietic activity and a minor modulation of the gut microbiome; however, FFT did not enhance growth nor improve survival of post-weaning piglets.

Finally, the article by Tanghe et al., evaluated if the supplementation of a mixture of specific fiber fractions derived from Araceae root and citrus for 14 days could prevent *E. coli* adhesion and the subsequent immune responses in post-weaning piglets. The authors demonstrated that the supplemented mixture reduced *E. coli* adhesion to the small intestine, reducing the potential pathogen progress, and stimulated SCFAs producing bacteria in the large intestine. The reduced *E. coli* adhesion was also reflected in lower intestinal inflammation, seen as a decrease in fecal myeloperoxidase, a specific marker of neutrophil activity.

Overall, considering the ability of nutritional and feeding strategies to influence the microbiota, intestinal health, and, consequently, the immune response, they also contribute to energy partition and growth. Therefore, the importance of the first days of life in driving the response of the animals in the long term; nutritional interventions focused on this productive period are becoming increasingly relevant and promising to improve the robustness of piglets. Thus, the ongoing collaboration between leading experts in diverse fields, namely nutrition, microbiology, and immunity, and their exchange with the industry and other relevant stakeholders are encouraged to facilitate the transformation of the swine-rearing system into a more ethical and sustainable one.

## Author contributions

All authors listed have made a substantial, direct, and intellectual contribution to the work and approved it for publication.

## References

[1] Stygar AH Chantziaras I Maes D Aarestrup Moustsen V De Meyer D Quesnel H. Economic feasibility of interventions targeted at decreasing piglet perinatal and pre-weaning mortality across European countries. Porcine Health Manag. (2022) 8:22. 10.1186/s40813-022-00266-x 35650652 PMC9158370

[2] Blavi L Solà-OriolD Llonch P López-Vergé S Martín-Orúe SM Pérez JF. Management and feeding strategies in early life to increase piglet performance and welfare around weaning: a review. Animals. (2021) 11:302. 10.3390/ani11020302 33503942 PMC7911825

[3] Missotten J Michiels J Ovyn A De Smet S Dierick N. Fermented liquid feed for weaned piglets: impact of sedimentation in the feed slurry on performance and gut parameters. Czech J Anim Sci. (2015) 60:195–207. 10.17221/8169-CJAS

[4] Pedersen A. Fermenteret Vådfoder Til Smågrise. Report 510. Landsudvalget for Svin (2001). Available online at: https://svineproduktion.dk/publikationer/kilder/lu_medd/medd/510

[5] Ott SJ Waetzig GH Rehman A Moltzau-Anderson J Bharti R Grasis JA. Efficacy of sterile fecal filtrate transfer for treating patients with clostridium difficile infection. Gastroenterology. (2017) 152:799–811.e7. 10.1053/j.gastro.2016.11.010 27866880

[6] Rhouma M Fairbrother JM Beaudry F Letellier A. Post weaning diarrhea in pigs: risk factors and non-colistin-based control strategies. Acta Vet Scand. (2017) 59:31. 10.1186/s13028-017-0299-7 28526080 PMC5437690

